# Typhlitis—Lachesis 2c

**Published:** 1898-05

**Authors:** J. Fitz-Mathew

**Affiliations:** West Sound, Washington


### TYPHLITIS—LACHESIS 2C.

J. Fitz-Mathew, M. D., West Sound, Washington.

Delicate school-boy, aged ten years, had been drinking bad water, eating the green tops of some plant, and violently exercising at play. Symptoms: Frequent vomiting, yellowish, greenish, preceding; patient on his back, characteristic posture; right leg drawn up; erection of penis; hard swelling in right or ileo-inguinal region, intensely painful to touch or movement; temperature, 103; pulse, 130 to 140; tongue pointed, bright scarlet; always worse after sleep; would wake up, and then scream with pain. R. Lachesis 2c (Dunham); second dose in half an hour; then every hour till improvement was evident; Sac-lac. to follow. Four doses were given. I found patient in the morning wonderfully better and out of danger. Later an enema of warm water removed a mass of very fœtid fecal matter. Patient was now convalescent.

